# Comprehensive Knowledge of HIV and AIDS and Related Factors in Angolans Aged between 15 and 49 Years

**DOI:** 10.3390/ijerph20196816

**Published:** 2023-09-23

**Authors:** Neida Neto Vicente Ramos, Inês Fronteira, Maria do Rosário O. Martins

**Affiliations:** 1 Global Health and Tropical Medicine, Institute of Hygiene and Tropical Medicine, NOVA University of Lisbon, 1249-008 Lisbon, Portugal; 2 Comprehensive Health Research Center, National School of Public Health, NOVA University of Lisbon, 1249-008 Lisbon, Portugal

**Keywords:** Angola, HIV, AIDS, comprehensive knowledge, health literacy

## Abstract

A comprehensive knowledge of HIV and AIDS among men and women in Africa is reportedly low. To the best of our knowledge, no studies using any definition of comprehensive knowledge of HIV and AIDS have been conducted in Angola. To address this gap, we aimed to describe the comprehensive knowledge held by individuals aged between 15 and 49 years regarding HIV and AIDS and some associated factors, using the most recent Angolan demographic and health survey (DHS). Using an observational, cross-sectional design, we analyzed data collected from 19,785 individuals aged between 15 and 49 years for the 2016 DHS in Angola. We conducted a logistic regression analysis of descriptive and complex samples to examine the data and to unravel possible factors associated with having a comprehensive knowledge of HIV and AIDS. Almost half of the respondents (47.7%) had a general comprehensive knowledge of HIV and AIDS. Individuals who watched television (adjusted odds ratio [aOR]: 2.40; 95% CI: 2.11, 2.72) or read newspapers and magazines (aOR: 1.99; 95% CI: 1.72, 2.30) more than once a week had higher odds of having a comprehensive knowledge of HIV and AIDS compared to those who did not. Similarly, having completed primary education and above (aOR: 1.83; 95% CI: 1.67, 2.00) or living in urban areas (aOR: 1.51; 95% CI: 1.34, 1.71) increased the likelihood of individuals having a comprehensive knowledge of HIV and AIDS compared to their counterparts. These results reflect inequalities that require further attention at either a research or a political level. Nevertheless, we consider that these results can assist decision-makers in advocating for continuous investment in HIV health literacy and in adapting global solutions to local Angolan contexts.

## 1. Introduction

The African region is more burdened by the human immunodeficiency virus (HIV) than other regions of the world [1]. Around 16% of the world’s new HIV infections and 21% of global AIDS-related deaths occur in the African region [2].

HIV and AIDS and other sexually transmitted diseases (STDs) are the leading cause of death among adolescents and young adults in sub-Saharan Africa (26.12 deaths per 100,000 inhabitants) [1]. HIV increases health and social vulnerability and has a significant impact on health status, social relationships, and well-being [3]. A number of studies have reported that, in sub-Saharan Africa, HIV and AIDS are associated with low levels of health literacy [4,5,6] which, by the same token, determine low levels of adherence to the antiretroviral treatment available [4,7,8].

In 2020, the UNAIDS estimated the prevalence of HIV-infection in Angola to be 1.8%. In that same year, 340,000 children and adults were living with HIV, with an incidence of 22,000 new infections per year, and 33% of those infected were receiving antiretroviral treatment [9]. In Angola, a greater prevalence of HIV infection is found in female sex workers (8%) and men who are in prison (15%) [9]. If preventive measures are not strengthened, this increased prevalence in high-risk groups has the potential to spread to the general population [1,10].

The prevalence of HIV in Angola is expected to increase in the near future due to the socio-political and economic factors affecting the continent and the country [11]. Moreover, population flows between Angola and neighboring countries such as Namibia and Zambia, both of which have a higher prevalence of HIV, can further aggravate the epidemy in the country. Additionally, the chronicity of HIV infection and its treatment (i.e., strict medical follow-up with an emphasis on correct adherence to treatment plans and the importance of following a regular lifestyle and avoiding risky behaviors) is paramount to a better and longer life expectancy, making the survival of people living with HIV and their control over the condition dependent on their knowledge of the disease, its mechanisms of transmission, and available treatments so that they may make the best decisions regarding their health [12,13].

This study’s conceptual framework aligns with the health belief model (HBM), which suggests that an individual’s behavior is determined by their perception of the potential threat of a disease and the perceived advantages of adopting preventive measures. This framework is reliable and has been used in previous studies on HIV and AIDS knowledge [14].

The concept of comprehensive knowledge of HIV and AIDS includes an understanding of the transmission of HIV and the ability to refute misconceptions about HIV and AIDS [10]. Although this concept can vary between studies and regions [10,15,16,17], it is widely accepted that having a comprehensive knowledge of HIV can be the first step toward HIV-testing, modifying unsafe behaviors among populations at risk, and the acceptance of treatment [11].

Many different factors seem to play a role in the disparity between the number of people diagnosed with HIV and the number of people who achieve viral suppression and greater longevity [13,18]. These include education, access to health care services, literacy, family and social support, the availability of antiretroviral drugs, treatment costs, adherence to HIV treatment, stigma and discrimination, substance abuse, mental illness, and relationships with health professionals [3,13,19,20,21]. As such, inadequate knowledge about HIV may be a significant and relevant factor contributing to the transmission of HIV and poor antiretroviral treatment outcomes [7,22,23] through stigma, a poor capacity for prevention, decreased motivation to seek health services, a low uptake of HIV testing, the postponement of treatment, and poor adherence to treatment. In sub-Saharan Africa, specific circumstances like early marriages, gender violence and discrimination, inadequate access to reproductive health services, and civil conflicts have induced gender imbalances regarding the risk of HIV infection, taking a higher toll on females [8,24,25,26]. This is worrying as the comprehensive knowledge of HIV and AIDS among men and women in Africa is reportedly low [8,27,28].

Obtaining accurate and comprehensive information regarding the levels of knowledge about HIV and AIDS from credible sources can be the best approach to understanding disparities and vulnerabilities [8,15] and to promoting focused HIV and AIDS education and the delivery of practical messages to protect vulnerable groups.

It is important to study these factors as they may influence knowledge levels and support campaigns aimed at increasing general knowledge of HIV and AIDS and health literacy in general.

To the best of our knowledge, no studies using any definition of comprehensive knowledge about HIV and AIDS have been conducted in Angola. To address this gap, we aimed to describe the comprehensive knowledge held by individuals aged between 15 and 49 years regarding HIV and AIDS and some associated factors, using the most recent Angolan demographic and health survey (DHS).

## 2. Methods

We conducted a correlational and cross-sectional study using secondary data collected for the first DHS questionnaire in Angola between November 2015 and February 2016 [29]. This is the only DHS conducted in Angola so far. Angola is a developing lower-middle-income economy in sub-Saharan Africa [30]. The country has approximately 30 million inhabitants [31], 67% of whom live in urban areas [29,31]. With a fragile and under-resourced health system [32], the country has a current health expenditure of 3.0% of its gross domestic product (GDP) [33].

Access to the DHS database and permission to use the data was free of charge and was granted to the authors upon request to the DHS program. In Angola, DHSs are financed by the United States Agency for International Development (USAID) and implemented by the National Institute of Statistics in Angola (INE) in cooperation with the Angolan Ministry of Health (MINSA) [34].

The DHS Program has a worldwide reputation for collecting and disseminating accurate, nationally representative data on fertility, family planning, maternal and child health, gender, HIV and AIDS, malaria, and nutrition [35]. The DHS surveys are reliable and valid, and they are conducted well using standard procedures: questionnaires are confirmed when they first arrive from the field to ensure the correct numbers of questionnaires and the selection of eligible respondents [35]; all questionnaires are checked after data-entry to guarantee that the expected data were, in fact, entered [35]; and all questionnaires are entered twice and verified via a comparison of both datasets. All discrepancies are resolved [35].

The inputted data are checked for inconsistencies and, where possible, inconsistencies are resolved. Some missing data are imputed where possible. A set of quality control tables is generated on a frequent basis [35].

In fact, the DHS is a reliable source of data, and numerous African studies have validated this survey to measure comprehensive knowledge about HIV and AIDS [8,15,16,24,27,28]. Further details on the method of data collection and the questionnaires used can be found in the DHS Angola 2015 report [29].

The population targeted by the Angolan DHS were female and male individuals aged between 15 and 49 years or 15 and 55 years, respectively. These age groups are often used because they represent the population who are of fertile age and are thus good proxies for the sexually active population.

The data from the DHS dataset allowed us to have a representative sample (at the provincial level) of the targeted population in Angola that was up to date (the data were collected in 2015) [29].

The sample used in our study included male and female individuals aged 15 to 49 years, totaling 19,785 individuals. Males older than 49 years were excluded from our sample to control for the effect of possible age differences between the two genders.

Variables:

The outcome variable was comprehensive knowledge of HIV and AIDS (Yes/No), defined by the sum of points (less than four points for no; four or five points for yes) obtained by adding one point per correct answer and zero points per wrong answer to each of the following questions in the DHS [35]:

(i) Can you get HIV from a mosquito bite?
(ii) Can we reduce the chance of HIV by always using condoms correctly during sex?
(iii) People can get HIV if they share food with someone infected with HIV?
(iv) A healthy-looking person can have HIV/AIDS?
(v) Can we reduce the chance of HIV by only having one sex partner without HIV?

This method was previously used in the DHS, Millennium Development Goals, and Sustainable Development Goals [36,37].

Ten independent variables were included in the analysis according to previous African studies [4,8,15,16,38], namely, gender, age, marital status, province of residence, region, education level, frequency of reading a newspaper or magazine, listening to the radio, and watching television, and language spoken at home.

Data Analysis:

We proceeded with the DHS recommendation [8] to calculate a WEIGHT variable to make the data more accurate.

The analysis was carried out in three steps:

I. In the first step, we computed the comprehensive knowledge of HIV and AIDS variable.
II. In the second, step we used the chi-square test/Fisher’s exact test to analyze the associations between having comprehensive knowledge of HIV and AIDS and each independent variable.
III. Then, we performed a multivariable logistic regression to determine associations. The relationship between the predictor and outcome variables was estimated using odds ratio (ORs) with a 95% confidence interval (CIs).

The IBM Statistical Package for the Social Sciences (SPSS) 25.0 for Windows was used to do the computation.

Ethical issues:

This study does not place the dignity of human beings or animal species at risk. Aspects such as confidentiality and the anonymity of the respondents’ identities were considered and preserved in the primary source. This study used a secondary data analysis and therefore no additional approval was required since the data are accessible in the public domain at https://dhsprogram.com/.accessed on 3 April 2022.

## 3. Results

### 3.1. Sociodemographic Characteristics of Respondents

Of the 19,785 subjects included in the study, more than half were female (73%) and under 30 years of age (60%), with the most frequent age group being individuals aged 15–19 years old (25%). Most of the respondents had completed primary school (66%), spoke Portuguese at home (72%), lived in an urban area (70%), and were not married (88%), and 40% were from Luanda.

Concerning the use of mass media, 48.5% of the respondents prefer to watch television at least once a week, 39.4% do not listen to the radio or listen to it less than once a week (31.2%). About 33.1% do not read newspapers and/or magazines or read them less than once a week (24.3%). The Angolan provinces most represented in our study were Luanda (39.6%), Benguela (8.1%), Huila (8%), Kwanza Sul (6.8%), and Huambo (6.4%) (Table 1 summarizes the sociodemographic characteristics of respondents; see Table 1).

### 3.2. Comprehensive Knowledge of HIV and AIDS in Angola

According to our findings, in Angola, 47.7% of individuals aged 15–49 years had a comprehensive knowledge of HIV/AIDS, i.e., they correctly answered at least four of the five questions related to having a comprehensive knowledge of HIV and AIDS.

**Figure 1:**
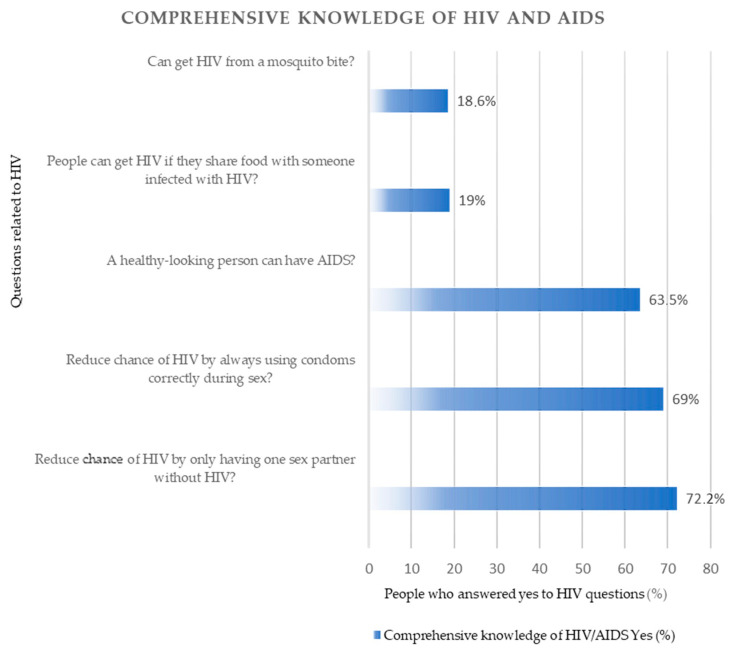
The distribution of people who answered “yes” to the five questions related to HIV/AIDS.

Figure 1 shows the distribution of people who answered “yes” to the five questions related to having a comprehensive knowledge of HIV and AIDS.

### 3.3. Factors Associated with Having a Comprehensive Knowledge of HIV and AIDS in Angola

All variables, such as sex, marital status, education level, area of residence, speaking Portuguese at home, listening to the radio, watching television, and reading newspapers or magazines more than once a week, were analyzed using the chi-square test (X^2^) and were associated (*p* < 0001) with having a comprehensive knowledge of HIV and AIDS (Table 2).

The predictors for having a comprehensive knowledge of HIV and AIDS in Angola in our study were watching television, reading journals and magazines, education level, place of residence, and the language spoken at home. This shows that there is a significant relationship between the factors mentioned above and having a comprehensive knowledge of HIV and AIDs (*p* < 0001).

Watching Television and Reading Journals or Magazines:

The logistic regression analysis revealed that the odds of having a comprehensive knowledge of HIV and AIDS were higher among individuals who watch television (adjusted odds ratio (aOR): 2.40; 95% confidence interval (CI): 2.11, 2.72) or read journals and magazines (aOR: 1.99; 95% CI: 1.72, 2.30) more than once a week, compared to those who do not watch television at all or do not read journals or magazines at all.

Education:

People who had completed a primary level of education and above were more likely to have a comprehensive knowledge of HIV and AIDS (aOR: 1.83; 95% CI: 1.67, 2.00) when compared to those who had not completed a primary level of education.

Place of Residence:

People living in urban areas (aOR: 1.51; 95% CI: 1.34, 1.71) or those who speak Portuguese at home (aOR: 1.40; 95% CI: 1.24, 1.56) were more likely to have a comprehensive knowledge of HIV and AIDS compared to those who live in rural areas and those who speak local languages at home.

In our logistic regression analysis, gender, marital status, age group, and listening to the radio were not associated (*p* > 0.05) with the odds of having a comprehensive knowledge of HIV and AIDS (Table 2 summarizes the results of the chi-square and multivariate logistic regression analyses of the predictors for having a comprehensive knowledge of HIV and AIDS in Angola).

## 4. Discussion

In Angola, where the majority of the population is young and has limited financial resources and low levels of education, it is particularly important to assess the comprehensive knowledge of HIV and AIDS [29,34]. There is also a significant prevalence of sexually transmitted infections such as syphilis and hepatitis B and C in the Angolan population [39,40].

Our study addressed the prevalence of comprehensive knowledge of HIV and AIDS and some related factors using national representative data from the Angolan DHS. Even though some individuals demonstrated a fair knowledge when answering single questions related to HIV and AIDS, the same was not true when the indicator of comprehensive knowledge that included five questions was considered.

Similar to findings reported in other studies that used the DHS, we found a predominance of females in our sample, which is explained by the focus of the DHS on maternal and child health [41]. Nevertheless, women of childbearing age are frequently used as proxies for the sexually active population, which is known to be at a higher risk of HIV and AIDS and other sexually transmitted infections [42].

Our results suggest that the prevalence of comprehensive knowledge of HIV and AIDS in the Angolan population aged from 15 to 49 years is only 48%. This finding is similar to the results described for Mozambique (42%) and Malawi (43%) [15,41] and is supported by a recent literature review showing that the level of comprehensive knowledge of HIV and AIDS in sub-Saharan Africa is still low [17,27]. This demonstrates that the current percentage of young people in Africa who have access to reliable information and necessary services to increase their life skills and decrease their risk of HIV infection falls considerably short of the target set by the United Nations General Assembly Special Session (UNGASS) in 2001, which aimed to ensure that 95% had such access worldwide [26,43].

In our study, we found that the prevalence of comprehensive knowledge of HIV and AIDS in women was 44%, slightly higher than the 37% described by Teshale et al. for women from 15 sub-Saharan African countries, although it is lower than in Rwanda (66%) [8]. For males, the prevalence of comprehensive knowledge of HIV and AIDS was 55%, a number very similar to that estimated for men in 29 sub-Saharan African countries (51%), but also lower than in Rwanda (76%) [28]. Cultural norms and values that discourage women from discussing sex-related issues, while encouraging men to do so, may explain the gender imbalance in the knowledge of HIV and AIDS [44]. This finding highlights the need for further efforts to achieve gender equality in education and knowledge, particularly in the health sector. This is of concern because women bear the burden of educating and caring for their children and therefore need to have adequate knowledge of fundamental health issues.

In Angola, the 27-year military conflict may have had a major impact on learning, educational outcomes, and general knowledge. During the conflict, girls living in affected areas were less likely to complete their compulsory education than girls of the same age living in regions that were not directly affected by the conflict, such as Luanda [25].

Our analysis did not show a statistical association between gender and marital status to predict having a comprehensive knowledge of HIV and AIDS. However, this finding is not consistent with other African studies that suggest that married individuals have a greater comprehensive knowledge of HIV and AIDS [15,27,45].

Individuals living in an urban area and those who had completed primary school had higher odds of having a comprehensive knowledge of HIV and AIDS than their counterparts. This result is supported by other studies [8,15,16,17,41] and might suggest a necessity to increase equality in healthcare access, especially with respect to health information. It is crucial that less-educated individuals residing in rural areas have as much access to information and health services as their urban counterparts. Communication methods should be designed to improve the transmission and comprehension of information about HIV that is adjusted to the sociocultural level of the intended audience [46]. This information should be disseminated in places such as churches, healthcare facilities, and supermarkets, which cater to these individuals’ regular routines.

Although communication plays an active role in the acquisition of knowledge [47], in our study, listening to the radio was not associated with having a comprehensive knowledge of HIV and AIDS, which contradicts previous findings from Malawi and 15 sub-Saharan African countries [8,17]. Over six thousand individuals reported not utilizing mass media, including television, radio, and newspapers. Thus, this prompts an inquiry as to whether this scenario is due to the decisions of individuals, or if it arises from restricted accessibility to these media because of geographic or economic factors or a lack of trust or interest. Nevertheless, it is challenging to guarantee that information regarding the prevention and transmission of HIV and AIDS can successfully reach these individuals.

Higher odds of having a comprehensive knowledge of HIV and AIDS were found among respondents who watch TV and read newspapers and magazines more than once a week, consistent with previous findings in global studies [48,49,50]. Again, the use of mass media in Angola could possibly be affected by economic factors which determine the presence of these media in the home, and by the distribution of electricity [51]. Nevertheless, mass media, such as newspapers, television and radio, are of great importance in Angola as they raise awareness about sexual health by continually repeating messages about the transmission of HIV and AIDS and by advising the population about the risks and complications of the disease [52]. More television programs on health-promotion should be offered to the Angolan population and promoted in local languages and rural areas.

Improving health literacy, promoted by trusted media, is important for reducing misinformation and related problems and is a key step toward achieving at least three sustainable development goals (SDGs), namely, SDG 3, to ensure health and well-being for all, including a bold commitment to end the epidemics of AIDS, tuberculosis, malaria, and other communicable diseases by 2030; SDG 4, to ensure inclusive and equitable quality education and promote lifelong learning opportunities for all by 2030; and SDG 5, to achieve gender equality and empower all women and girls.

This research demonstrates the current state of the art of comprehensive knowledge about HIV and AIDS in adolescents and adults in Angola, a sub-Saharan African country, making evident some factors that may compromise the achievement of the 2030 Agenda for Sustainable Development.

## 5. Limitations

Our study had a few significant limitations: firstly, the data were limited to the 2015 and 2016 Angolan DHSs. The cross-sectional nature of this study did not permit for causality to be inferred from the findings. Despite their limitations in data exploration, demographic and health surveys are credible sources of information from which data can be extracted to enable us to analyze the epidemiological and health characteristics of a population.

## 6. Conclusions

Despite the existence of some studies using DHS data for analyzing knowledge about HIV and AIDS, research gaps remain in sub-Saharan Africa. This study contributes by filling a gap in the literature relating to comprehensive knowledge of HIV and AIDS in Angola. Using national representative data, this study demonstrates the prevalence of comprehensive knowledge of HIV and AIDS and its related factors. Even though the respondents demonstrated good knowledge when answering each individual question related to HIV and AIDS, the same was not true when the five questions were analyzed together.

Our research identifies the characteristics of the Angolan population with the lowest level of comprehensive knowledge of HIV and AIDS and highlights existing and persistent inequalities in Angola.

This study also emphasizes the possible relevance of television campaigns in improving the comprehensive knowledge of HIV and AIDS, as other media tend to be used less by Angolans.

These findings can help health decision-makers adjust strategic interventions regarding HIV and other health issues in Angola. In addition, they can serve as a wake-up call for governments and civil society to focus on investing more in health education for children, young people of all genders, less-educated women, and rural residents, thus targeting inequalities and increasing the focus on health literacy for better health outcomes. Education regarding HIV-prevention needs to be broadened and adjusted for everyone to enhance the comprehensive understanding of the disease. Tailored campaigns should be conducted specially for vulnerable communities to prevent misinformation and encourage safe behaviors.

## Figures and Tables

**Table 1:** Sociodemographic characteristics of respondents, with weighted frequencies and percentages.

	Sociodemographic Characteristics of Respondents (n = 19,785)		
Variables	Categories	Weighted Frequency	Weighted Percentage (%)
Gender	Male	5418	27.4
	Female	14,360	72.6
Age groups	15–19	4925	24.9
	20–24	4070	20.6
	25–29	3364	17
	30–34	2394	12
	35–39	2021	10
	40–44	1698	8.6
	45–49	1311	6.6
Marital status	Not married	17,436	88.1
	Married	2349	11.9
Residence	Urban	13,916	70.3
	Rural	5869	29.7
Region	Cabinda	481	2.4
	Bengo	225	1.1
	Zaire	414	2.1
	Uíge	965	4.9
	Luanda	7828	39.6
	Cuanza Norte	229	1.2
	Cuanza Sul	1355	6.8
	Malange	616	3.1
	Lunda Norte	485	2.5
	Lunda Sul	312	1.6
	Benguela	1608	8.1
	Huambo	1271	6.4
	Bié	795	4
	Moxico	350	1.8
	Huíla	1574	8
	Namibe	245	1.2
	Cuando Cubango	329	1.7
	Cunene	703	3.6
Education	Complete primary and more	13,116	66.3
	Incomplete primary	6669	33.7
Radio	At least once a week	5826	29.4
	Less than once a week	6173	31.2
	Not at all	7786	39.4
Television	At least once a week	9589	48.5
	Less than once a week	3859	19.5
	Not at all	6336	32
Journals and magazines	At least once a week	1617	8.2
	Less than once a week	4802	24.3
	Not at all	6547	33.1
Language spoken at home	Portuguese	14,171	71.6
	Local languages	5614	28.4

**Table 2:** Chi-square and multivariate logistic regression analyses of factors associated with comprehensive knowledge of HIV and AIDS in Angola.

Variables	Categories	Chi-Square Analysis		Multivariate Logistic Regression Analysis	
		Weighted Frequency (N = 19,785	Weighted Percentage (%)	Crude OR (CI 95%)	Adjusted OR (CI 95%)
Gender	Male	2989	55.2	1.51 (1.42–1.61)	0.90 (0.91–1.07)
	Female	6449	44.9	ref	ref
Age groups	15–19	2439	49.5	1.34 (1.21–1.54)	0.93 (0.79–1.11)
	20–24	1995	49	1.34 (1.18–1.51)	0.913 (0.76–1.09)
	25–29	1961	50.3	1.40 (1.24–1.60)	1.04 (0.86–1.25)
	30–34	1152	48.1	1.29 (1.12–1.47)	1.14 (0.93–1.38)
	35–39	903	44.7	1.12 (0.98–1.29)	1.20 (0.98–1.47)
	40–44	710	41.8	1.000 (0.86–1.16)	0.95 (0.77–1.18)
	45–49	548	41.8	ref	ref
Marital status	Not married	8433	48.4	0.80 (0.73–0.87)	1.13 (0.99–1.28)
	Married	1005	42.8	ref	ref
Residence *	Urban	8236	59.2	5.63 (5.24–6.05)	1.51 (1.34–1.71)
	Rural	1202	20.5	ref	ref
Education *	Completed primary education and above	392	55	2.44 (2.30–2.60)	1.83 (1.67–2.00)
	Did not complete primary education	94	33.3	ref	ref
Radio	More than once a week	176	62.5	3.51 (3.27–3.77)	1.09 (0.97–1.21)
	At least once a week	241	53.2	2.39 (2.23–2.56)	1.09 (0.98–1.21)
	Not at all	5593	32.2	ref	ref
Television *	More than once a week	99	67.7	8.35 (7.74–8.99)	2.40 (2.11–2.72)
	At least once a week	334	43.3	3.04 (2.78–3.32)	1.32 (1.16–1.52)
	Not at all	202	20.1	ref	ref
Newspapers and magazines *	More than once a week	170	77.9	3.47 (3.06–3.94)	1.99 (1.73–2.30)
	At least once a week	107	69.9	2.29 (2.12–2.43)	1.626 (1.49–1.77)
	Not at all	456	50.3	ref	ref
Language spoken at home *	Portuguese	258	57.5	4.53 (4.22–4.86)	1.39 (1.24–1.56)
	Local languages	100	23	ref	ref

Ref = reference category. * (*p* < 0001) in logistic regression.

## Data Availability

Data supporting the reported results are available upon request from the DHS program, which can be found on the web page of The Demographic and Health Surveys Program at https://dhsprogram.com/data/dataset/Angola_Standard-DHS_2015.cfm?flag=0. Accessed on 3 April 2022.
